# A Simple High-Throughput Method for the Analysis of Vicine and Convicine in Faba Bean

**DOI:** 10.3390/molecules27196288

**Published:** 2022-09-23

**Authors:** Aaron C. Elkins, Simone J. Rochfort, Pankaj Maharjan, Joe Panozzo

**Affiliations:** 1 Agriculture Victoria, AgriBio, Centre for AgriBioscience, Bundoora, VIC 3083, Australia; 2 School of Applied Systems Biology, La Trobe University, Bundoora, VIC 3083, Australia; 3 Agriculture Victoria, Grains Innovation Park, Horsham, VIC 3400, Australia; 4 Centre for Agriculture Innovation, University of Melbourne, Parkville, VIC 3010, Australia

**Keywords:** vicine, convicine, faba bean, anti-nutritional, quantitation, LC-MS

## Abstract

The faba bean is one of the earliest domesticated crops, with both economic and environmental benefits. Like most legumes, faba beans are high in protein, and can be used to contribute to a balanced diet, or as a meat substitute. However, they also produce the anti-nutritional compounds, vicine and convicine (v-c), that when enzymatically degraded into reactive aglycones can potentially lead to hemolytic anemia or favism. Current methods of analysis use LC-UV, but are only suitable at high concentrations, and thus lack the selectivity and sensitivity to accurately quantitate the low-v-c genotypes currently being developed. We have developed and fully validated a rapid high-throughput LC-MS method for the analysis of v-c in faba beans by optimizing the extraction protocol and assessing the method of linearity, limit of detection, limit of quantitation, accuracy, precision and matrix effects. This method uses 10-times less starting material; removes the use of buffers, acids and organic chemicals; and improves precision and accuracy when compared to current methods.

## 1. Introduction

The faba bean (*Vicia faba* L.) is a nitrogen-fixing legume that has both economic and environmental benefits. In Australia, 2.3 million tons of different pulses, including faba bean, field pea (*Pisum satvium* L.), chickpea (*Cicer arietinum* L.), lentils (*Lens culinaris* Medik.), Australian sweet lupin (*Lupinus angustifolius* L.) and mungbean (*Vigna radiata* L.), were produced annually from 2009 to 2015 with primary production in South Australia, Victoria and New South Wales [1]. Globally, in 2016, 4.5 million tons of faba bean crop was cultivated, with China, Ethiopia, and Australia being the main producers [2].

One of the earliest domesticated crops, faba beans have a long history of being used for human consumption [2,3], but they are also commonly used as a crop for animal feed, foraging, and medicine, and are one of the most versatile globally produced crops [2]. The consumption of faba beans, like most pulses, contributes to a balanced diet, due to the high protein content of 26–38% of the seed [1,4,5,6], which is also high in lysine, carbohydrates [1,5], fibre, and phytochemicals [5].

Despite the potential benefits, faba beans also produce anti-nutritional compounds that can have adverse effects by reducing nutrient digestibility [3], limiting their use in food and feed formulations [1]. Vicine (2,6-diamino-4,5-dihydroxypyrimidine-5-β-D-glucopyranoside) and convicine (2,4,5,trihydroxy-6-aminopyrimidine-5-β-D-glucopyranoside) are the two major anti-nutritional compounds found in faba beans, with levels varying depending on cultivar, maturation, cultivation climate, and soil properties [3,7]. Vicine and convicine (v-c) are reported to be synthesised in the testa during the seed filling stage [2,8,9], before being transported to the cotyledon, where they accumulate into a concentration of 6–14 g/kg [1,2,5,10].

Once consumed, v-c are enzymatically degraded by the β-glucosidase enzyme in the small intestine to the reactive aglycone divicine (2,6-diamino-4,5-dihydroxypyrimidine) and isouramil (6-Amino-2,4,5-trihydroxypyrimidine) [1,2,4,5,6,11,12,13]. Accumulation of the aglycones can potentially be toxic to individuals with a genetic deficiency of glucose-6-phophate dehydrogenase, leading to haemolytic anaemia or favism [1,3,4,5,10,11,14]. Favism affects approximately 400 million people globally [1,4,11], with the highest prevalence in Asia, the Mediterranean and Africa [6,11].

Concentrations of v-c in faba bean seeds and flour can be reduced or eliminated by roasting, boiling, or microwaving. Furthermore, soaking in water, a weak acid, alkaline solution, or fermentation [1,2,5,6,10] prior to consumption also reduces the risk of accumulating aglycones. These processes are important, as air classification during industrial scale processing has been shown to increase the concentration of v-c [1,2] up to 4-fold in the protein fraction [2].

The accurate determination of v-c in faba beans is especially important due to the increased interest in developing low-v-c cultivars. This research is underway, though not yet commercially available [1,2,4,5,6]. Current methods of extraction commonly involve long complex acid-extraction protocols using perchloric acid [1,4,5,6,10,13,14] and hydrochloric acid [3,13]; or large sample weights >1 g with an organic solvent such as methanol [10,12] or ethanol [6] and up to 50% water. Purves et al. performed a detailed extraction optimisation study comparing the responses of samples extracted with water; water with 1% formic acid; 70:30 acetone:water; 70:30 methanol:water; and 70:29:1 methanol:water:formic acid, finding that extraction with an organic solvent provided consistent results without the risk of continued biological activity that may occur in the water extract [4].

Analytical techniques used for the quantitation of v-c initially used spectrophotometry and colorimetry methods that were suitable at high concentrations [2,4]. Now, techniques predominantly employ liquid chromatography (LC) with UV detection [1,2,3,4,5,6,10,12,13,14], all of which lack the selectivity and sensitivity to accurately quantitate low levels of v-c [2]. Generally, methods use silica-based C_18_ columns for compound separation, and HILIC [4] and silica-based pentafluorophenylpropyl [1] columns have also been reported, although the long-term stability and reproducibility of the methods remain unclear. Utilising mass spectrometry (MS) provides greater selectivity and sensitivity, allowing for the accurate determination of cultivars with standard v-c levels, while also allowing for the accurate determination of low-v-c cultivars.

Despite the potential selectivity and sensitivity improvements, MS has predominantly been used as a confirmatory tool, complimenting UV quantitation for v-c [1,5,6,13]. Purves et al. used LC-MS to quantify the v-c concentration in 13 faba bean seeds, reporting significant improvements in selectivity and sensitivity compared to the UV analysis of the same samples, particularly for convicine [4]. The accurate measurement of v-c is essential to determine whether a crop is suitable for consumption; however, the current reported methods require improvement, especially for convicine [2].

Here, we report on a simple high-throughput method for the extraction and analysis of vicine and convicine in faba bean flour, improving on the previously reported method in terms of extraction simplicity, and the accuracy and precision of quantitation.

## 2. Results and Discussion

### 2.1. Extraction Optimisation

The extraction protocol was modified from those described in Purves et al., wherein five different extraction solvents were assessed. Purves et al. determined that a 70:30 acetone:water extraction solvent was optimal, as the method is also used for polyphenol extractions [4]. The study did note that the other organic solvents, including methanol, provided similar results to acetone. In a similar fashion, because 80% methanol is commonly used for the extraction of other metabolites, it was chosen for this study.

To optimise the extraction protocol, 10 mg of seven commercially available Australian faba bean cultivars (Farah, PBA Amberley, PBA Bendoc, PBA Marne, PBA Nasma, PBA Samira, and PBA Zahra) were extracted with 1 mL 80% methanol and the supernatant removed for analysis. This was repeated a further three times, providing four samples for analysis determine how many extraction steps are required. The EIC response for v-c in each of the four extraction samples was used to determine the extraction efficiency by dividing the response of each sample by the sum of all of the responses from samples 1–4 (Table 1). After two extraction steps, 99% of the total v-c was extracted from all seven samples, with extractions 3 and 4 contributing 0.4%. A two-step extraction with 80% methanol was adopted and validated.

### 2.2. Method Validation

#### 2.2.1. Linearity LOD and LOQ

**Figure 1:**
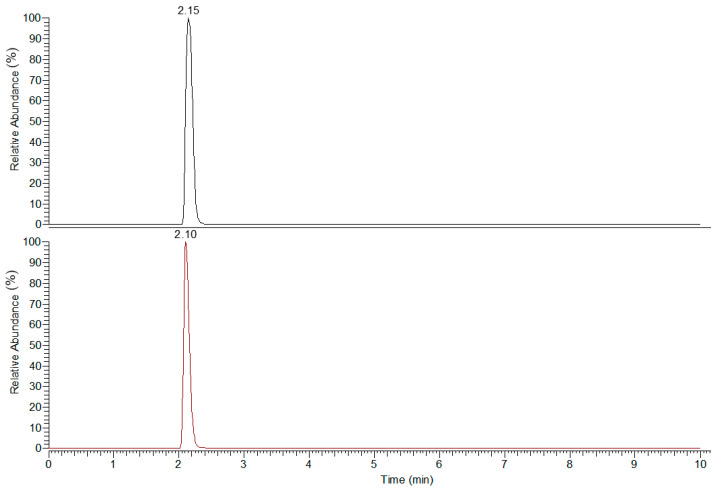
Extracted ion chromatogram of vicine and convicine.

A series of known concentration standards were prepared for v-c to determine linearity. The linear range of the method ranged from 12.5 to 2500 ng/mL and 5 to 1000 ng/mL convicine (Table 2). At higher concentrations, the responses were no longer linear (data not shown). Using a linear fit with no weighting, the R^2^ values for v-c were 0.9997 and 0.9984, respectively (Table 2). LOD and LOQ were calculated by using the data analysis tool in Microsoft Excel to obtain the standard error (SE) of the intercept. SE was then multiplied by 3.3 and 10, and divided by the slope of the curve, resulting in v-c LOD values of 0.0029 and 0.0028 mg/g; and a LOQ values of 0.0088 and 0.0084 mg/g, respectively (Table 2). These results are more consistent than the LOD and LOQ values reported by Purves et al. using selected reaction monitoring to target v-c [4]. SRM has a higher specificity for targeted compounds of interest; however, this is at the cost of identifying unknown compounds in the sample. Reported values of v-c in low genotypes range from 0.16–0.60 mg/g to 0.017–0.04 mg/g v-c [4], and are within the limit of quantitation of this method. No other LOD or LOQ data have been reported using LC-MS. The extracted ion chromatogram (EIC) of vicine and convicine is illustrated in Figure 1.

#### 2.2.2. Accuracy and Precision

The accuracy and precision of the method was determined by analysing five replicate injections of the standards at six concentrations (Table 3), and seven replicate extractions of the seven cultivars (Table 4). All v-c standards, except at or below LOD, showed excellent accuracy and reproducibility, significantly improving upon the values obtained by Purves et al. [4], especially at lower concentrations where a 4-fold improvement in precision was observed. Values obtained for convicine were slightly elevated compared to vicine, but these findings were consistent with Purves et al. [4].

The extraction and analysis method, described previously, was applied to seven replicate extractions of the seven cultivars to determine the suitability of the method. Seven replicate injections of the standard at high concentration and below the calculated LOD were also included to show the reliability of the method.

All samples were extracted with 2 × 1 mL 80% methanol and supernatants combined. A subsequent 1:20 dilution was then performed so samples would be in the linear range of the instrument. Undiluted samples were not analysed, however, can be used for low-v-c cultivars. The obtained values show excellent repeatability with RSD < 3.5% for all determinations, and <2.0% for v-c standards. Results for technical replicates have not been previously reported.

#### 2.2.3. Matrix Effect

To determine matrix effect, the extracted samples were spiked with a known concentration of the standard at the lower and upper limits of quantitation. Spikes at both levels were performed in triplicate, with the average concentration of the calculated amount in the sample divided by the expected concentration of the spike (Table 5). The results indicate that there is no matrix interference for vicine or for convicine at higher concentrations (97.9–102.6% for all samples). However, there may be some signal enhancement at low concentrations, particularly for convicine (114.0–132%). The method is suitable for analysis with minimal matrix interference.

#### 2.2.4. Method Comparison

The results obtained in the method validation were compared to previously published results by Skylas et al., wherein 10 cultivars were investigated for nutritional and anti-nutritional content (including v-c) [1]. Four varieties were used in this study: Farah, PBA Nasma, PBA Samira and PBA Zahra. These were common to those used by Skylas et al., although the growing locations (Charlick (Site 1) and Freeling (Site 2)) years were different.

Skylas et al. used the extraction protocol detailed by Marquadt and Frohlich [1,14]. Briefly, the method consisted of 100 mg of whole seed flour mixed with ammonium hydroxide and incubated for 1 hr at 70 °C. Samples were cooled and centrifuged with a 0.5 mL aliquot of the supernatant, mixed with 0.3 mL methanol and 0.3 mL chloroform. A 0.5 mL aliquot of the aqueous phase was dried overnight, then reconstituted in 0.1% formic acid and incubated for 10 min at 60 °C [1]. All samples were quantitated using UV (Table 6 Method 2). Samples were sourced from the 2016 and 2017 seasons, with the results averaged per site for comparison. Overall, the results obtained using this method (Table 6 Method 1) gave similar values to those obtained by Skylas et al. (Table 6 Method 2) across sites 1 and 2 [1], considering that the levels of v-c vary depending on stage of maturation, cultivation climate, and soil properties [3,7].

The concentrations determined using this method are also consistent with cultivars grown in Sweden [3], Finland [13], and Canada [4], wherein levels of v-c ranged between approximately 4.5–7.1 mg/g vicine and 1.2–4.6 mg/g convicine. Interestingly, no cultivar was common across all three sites for comparison; however, Gloria cultivar was grown in Canada and Sweden, resulting in significantly different v-c concentrations: 4.54 vs. 7.01 mg/g vicine and 1.62 vs. 1.91 convicine [3,4].

One limitation of this method is that low-v-c cultivars were unavailable to include. However, the undiluted extract will allow a 20-fold concentration equivalent to 0.005 and 0.004 mg/g v-c, respectively, well below reported the levels of vicine and convicine in low-v-c cultivars [4].

## 3. Materials and Methods

### 3.1. Reagents and Standards

All extraction and mobile-phase solvents were of HPLC grade. Methanol (≥99.9% pure), acetonitrile with 0.1% formic acid (≥98.5% pure), and water with 0.1% formic acid were purchased from Fisher Chemical (Fair Lawn, NJ, USA).

Vicine and convicine standards were purchased from Novachem Pty Ltd. (Heidelberg West, VIC, Australia) as the distributor for Toronto Research Chemicals (Toronto, ON, Canada). A stock solution of 25,000 ng/mL vicine and 10,000 ng/mL convicine was prepared using 10% methanol in water. Serial dilution was performed to prepare working standard solutions of 2500, 1250, 250, 125, 25 and 12.5 ng/mL vicine and 1000, 500, 100, 50, 10, and 5 ng/mL convicine in 80% methanol.

### 3.2. Sample Preparation

Seven commercially available Australian faba bean cultivars (PBA Amberley, PBA Bendoc, Farah, PBAMarne, PBA Nasma, PBA Samira and PBA Zahra) were sources from trials grown at Curyo, Victoria, Australia. The samples were milled to a homogenous powder of less than 0.5mm for analysis.

### 3.3. Extraction Optimisation

Each sample (10.0 + 0.2 mg) was weighed into an Axygen 2.0 mL microcentrifuge tube with analytical balance (Sartorius. MSU225S, Göttingen, Germany). Samples were extracted with 1 mL of 80% methanol (methanol and milli-Q water, 80:20, *v*:*v*), vortexed for 1 min (Ratek multitube vortex mixer, MTV1, Boronia, Victoria, Australia), sonicated for 5 min (SoniClean, 250TD, Thebarton, South Australia, Australia), and centrifuged at 13,000 rpm for 5 min (Eppendorf, 5415D, Hamburg, Germany). The supernatant was transferred to a pre-labelled 2.0 mL LC-MS vial for analysis. The pellet was re-extracted a further three times, with the supernatant transferred to an empty vial each time for analysis to determine extraction efficiency.

### 3.4. Method Validation

Samples were weighed and extracted as previously described. The supernatant was transferred to a second microcentrifuge tube, the pellet re-extracted a second time, and the supernatants combined. Samples were diluted 1:20 to fit within the linear range of the instrument. The method was validated for linearity; limit of detection (LOD); limit of quantitation (LOQ); accuracy; precision; repeatability; and matrix effect.

LOD and LOQ were determined by multiplying the standard error of the intercept—obtained using the regression data analysis tool in MS excel—by 3.3 for LOD and 10 for LOQ, then dividing the result by the slope of the curve.

Accuracy, precision, and repeatability of the standards were determined using the calculated concentration of the standards across five replicate injections compared to the expected concentrations. Extract repeatability was determined from comparing the results of seven replicate extractions of each cultivar, obtained as described above.

Matrix effect was determined by adding standards at two different levels—high (50,000 ng/mL vicine and 20,000 ng/mL convicine (HS)) and low (50 µL of 2500 ng/mL vicine 1000 ng/mL convicine (LS))—to different extracts of each of the seven cultivars. Each undiluted cultivar extract (50 µL) was combined with 50 µL of the high or low standard, and then made up to a final volume of 1 mL with 900 µL of 80% methanol.

### 3.5. Instrumentation and Data Analysis

Samples were analysed using a Thermo Scientific Vanquish ultra-high-performance liquid chromatography (UHPLC) system (Thermo Fisher Scientific, Bremen, Germany) coupled to a Thermo Fisher Q Exactive Plus mass spectrometer (QE MS) (Waltham, MA, USA; Thermo, Bremen, Germany). All MS data were acquired in positive electrospray ionization (ESI) mode over a mass range of 80–1200 m/z. The resolution was 35,000, the normalized collision energy was 30 V, and the maximum ion time was 200 milliseconds. The source heater temperature was maintained at 310 °C, and the heated capillary maintained at 320 °C. The sheath, auxiliary and sweep gases (N2) were 28, 15 and 4 units, respectively. Spray voltage was set at 3.6 kV. Prior to data acquisition, the system was calibrated with Pierce^®^ LTQ Velos ESI Positive Ion Calibration Solution (Thermo Scientific, product no. 88323).

Analytes were separated on a Phenomenex Synergi Polar-RP (150 mm × 2 mm, 4 μm) HPLC column with an isocratic mobile phase of 20% A (0.1% formic acid in water) and 80% B (0.1% formic acid in acetonitrile) over 4 min. The column was cleaned for 3 min at 100% B, before returning to the initial conditions for 3 min for re-equilibration and a total analysis time of 10 min. The flow rate of the method is 0.15 mL/min, with the column maintained at 40 °C.

All acquired data were quantitatively processed using Tracefinder 5.1 Build 110 (Thermo Fisher Scientific, San Jose, CA, USA).

## 4. Conclusions

The results reported herein display a fully validated rapid high-throughput LC-MS method for the analysis of v-c in faba beans. The method uses 10-times less starting material than the most commonly referenced method by Marquadt and Frohlich [14], streamlining the extraction protocol with reduced use of buffers, acids and organic chemicals, while improving the precision and accuracy of current methods. Low-v-c cultivars were not available to validate the method in low-v-c germplasm. However, using the undiluted extract demonstrated a 20-fold concentration equivalent to 0.005 and 0.004 mg/g v-c, respectively, well below the reported concentrations in low-v-c cultivars.

## Figures and Tables

**Table 1:** Summary of the extraction optimisation data from four consecutive extractions of the one sample and the sum of extracts 1 and 2 (∑).

	Vicine					Convicine				
	1	2	3	4	∑ *	1	2	3	4	∑ *
Farah	94.89	4.73	0.29	0.10	99.61	93.91	5.57	0.38	0.14	99.48
PBA Amberley	94.67	4.90	0.33	0.10	99.57	93.87	5.54	0.44	0.15	99.40
PBA Bendoc	94.48	5.11	0.32	0.09	99.59	93.10	6.35	0.43	0.12	99.45
PBA Marne	94.39	5.22	0.29	0.10	99.61	93.26	6.23	0.37	0.13	99.50
PBA Nasma	94.16	5.30	0.37	0.17	99.46	92.89	6.30	0.53	0.27	99.19
PBA Samira	94.86	4.71	0.33	0.11	99.56	93.67	5.74	0.44	0.16	99.41
PBA Zahra	95.11	4.39	0.38	0.12	99.50	93.94	5.33	0.53	0.20	99.27
Average	94.65	4.91	0.33	0.11	99.56	93.52	5.86	0.45	0.17	99.38
%RSD	0.35	6.66	10.80	23.62	0.06	0.46	7.14	14.71	30.71	0.11

* The sum of extraction step 1 and extraction step 2. Results reported as % total response of the four extracts. e.g., response 1/total response (extract 1–4).

**Table 2:** Retention time, linear range, limit of detection (LOD) and limit of quantitation (LOQ) of the method.

Standard	RT (min)	Concentration (ng/mL)	Equation	R^2^	LOD (mg/g)	LOQ (mg/g)
Vicine	2.15	12.5, 25, 125, 250, 1250, 2500	y = 69130.3x	0.9997	0.0029	0.0088
Convicine	2.10	5, 10, 50, 100, 500, 1000	y = 33538.0x	0.9984	0.0028	0.0084

**Table 3:** Accuracy and precision of the method comparing the mean concentration of the replicate injections (*n* = 5), the variation between injections (%RSD) and the difference between the calculated concentration of the standard and the expected concentration of the standard (% Conc. of Std) for vicine and convicine.

	**Vicine (ng/mL)**					
Mean conc.	12.60	25.20	125.40	249.20	1245.40	2504.20
%RSD	4.35	1.77	1.33	3.46	0.96	1.91
% Conc. of Std	100.80	100.80	100.32	99.68	99.63	100.17
	**Convicine (ng/mL)**					
Mean conc.	5.40	10.40	54.60	105.20	516.00	968.20
%RSD	10.14	5.27	2.46	4.91	2.59	3.47
% Conc. of Std	108.00	104.00	109.20	105.20	103.20	96.82

**Table 4:** Replicate analysis of seven commercially available Australian faba bean cultivars and two standards at the lower (Std 1) and upper (Std 2) limit of the linear range for vicine and convicine.

**Vicine**									
	**Sample (mg/g)**							**Standard (ng/mL)**	
**Replicate**	**Farah**	**PBA Amberley**	**PBA Bendoc**	**PBA Marne**	**PBA Nasma**	**PBA Samira**	**PBA Zahra**	**Std 1**	**Std 2**
1	4.90	5.08	3.89	4.03	5.22	5.67	4.25	24.91	1253.20
2	5.18	5.09	3.99	3.94	5.32	5.76	4.39	24.75	1240.94
3	4.97	4.90	4.06	3.97	5.57	5.83	4.21	24.65	1251.87
4	5.17	5.13	3.70	4.01	5.62	5.81	4.27	24.42	1246.61
5	4.86	5.25	4.04	4.02	5.15	5.81	4.12	24.01	1239.96
6	5.12	5.03	3.78	3.93	5.53	5.93	4.37	23.95	1243.86
7	4.94	5.07	3.93	3.72	5.27	5.80	4.09	24.40	1249.01
Average	5.02	5.08	3.91	3.94	5.38	5.80	4.24	24.44	1246.49
%RSD	2.66	2.07	3.46	2.66	3.48	1.36	2.69	1.48	0.42
**Convicine**									
	**Samples (mg/g)**							**Standard (ng/mL)**	
**Replicate**	**Farah**	**PBA Amberley**	**PBA Bendoc**	**PBA Marne**	**PBA Nasma**	**PBA Samira**	**PBA Zahra**	**Std 1**	**Std 2**
1	2.30	1.99	2.41	2.41	2.56	3.27	1.97	10.23	515.79
2	2.39	1.98	2.51	2.33	2.58	3.23	2.09	10.19	514.52
3	2.29	1.96	2.46	2.39	2.66	3.37	1.98	10.40	518.87
4	2.41	2.04	2.33	2.32	2.67	3.34	2.04	10.57	516.27
5	2.28	2.10	2.41	2.38	2.53	3.32	2.00	10.37	505.67
6	2.37	2.01	2.35	2.30	2.68	3.36	2.07	10.37	513.66
7	2.29	2.01	2.42	2.20	2.58	3.29	1.98	10.51	506.93
Average	2.33	2.01	2.41	2.33	2.61	3.31	2.02	10.38	513.10
%RSD	2.30	2.27	2.58	3.08	2.26	1.52	2.31	1.31	0.96

**Table 5:** Post extraction spikes to determine matrix effect.

	Vicine		Convicine	
	LS	HS	LS	HS
Farah	99.7	98.2	114.0	99.3
PBA Amberley	116.3	100.6	132.0	102.6
PBA Bendoc	100.3	99.2	112.0	101.0
PBA Marne	106.1	98.7	120.0	100.5
PBA Nasma	102.9	97.9	112.0	99.6
PBA Samira	109.9	98.9	130.7	99.2
PBA Zahra	92.5	98.0	102.7	102.0

LS = low spike; HS = high spike. Results reported as % calculated concentration divided by expected concentration.

**Table 6:** Result comparison of four faba bean cultivars grown in different regions determined by two different extraction and analysis methods.

	Vicine			Convicine		
	Method 1	Method 2 Site 1	Method 2 Site 2	Method 1	Method 2 Site 1	Method 2 Site 2
Farah	5.0	5.7	5.6	2.3	2.3	2.3
PBA Nasma	5.4	6.0	5.5	2.6	2.9	2.7
PBA Samira	5.8	6.1	5.9	3.3	3.1	3.0
PBA Zahra	4.2	4.8	4.7	2.0	2.1	2.0

Site 1 = Charlick South Australia; Site 2 = Freeling South Australia (Skylas et al. [1]).

## Data Availability

The raw data and processed data presented in this study are available on reasonable request from the corresponding author.

## Sample Availability

Samples of the compounds are not available from the authors.
